# Timing of appropriate empirical antimicrobial administration and outcome of adults with community-onset bacteremia

**DOI:** 10.1186/s13054-017-1696-z

**Published:** 2017-05-26

**Authors:** Ching-Chi Lee, Chung-Hsun Lee, Ming-Yuan Hong, Hung-Jen Tang, Wen-Chien Ko

**Affiliations:** 1 Division of Critical Care Medicine, Department of Internal Medicine, Madou Sin-Lau Hospital, No. 20, Lingzilin, 72152, Madou Dist, Tainan City, Taiwan; 2 Graduate Institute of Medical Sciences, College of Health Sciences, Chang Jung Christian University, Tainan, Taiwan; 3 Department of Internal Medicine, National Cheng Kung University Hospital, Tainan, Taiwan; 4 Department of Medicine, National Cheng Kung University Medical College, Tainan, Taiwan; 5 Department of Emergency Medicine, National Cheng Kung University Hospital, No. 138, Sheng Li Road, 70403 Tainan, Taiwan; 6 Department of Medicine, Chi-Mei Medical Center, Tainan, Taiwan; 7 Department of Health and Nutrition, Chia Nan University of Pharmacy and Science, Tainan, Taiwan; 8 Division of Infectious Disease, Department of Internal Medicine, National Cheng Kung University Hospital, No. 138, Sheng Li Road, 70403 Tainan, Taiwan; 9 Division of Infectious Disease, Department of Medicine, Chi-Mei Medical Center, No. 901, Chung-Hwa Road, Yung-Kang City, 710 Tainan Taiwan

**Keywords:** Initial antibiotic therapy, Inappropriateness, Bloodstream infection, Prognosis

## Abstract

**Background:**

Early administration of appropriate antimicrobials has been correlated with a better prognosis in patients with bacteremia, but the optimum timing of early antibiotic administration as one of the resuscitation strategies for severe bacterial infections remains unclear.

**Methods:**

In a retrospective cohort study, adults with community-onset bacteremia at the emergency department (ED) were analyzed. Effects of different cutoffs of time to appropriate antibiotic (TtAa) administration after arrival at the ED on 28-day mortality were examined, after adjustment for independent predictors of mortality identified by multivariate regression analysis.

**Results:**

Among 2349 patients, the mean (interquartile range) TtAa was 2.0 (<1 to 12) hours. All selected cutoffs of TtAa, ranging from 1 to 96 hours, were significantly associated with 28-day mortality (adjusted odds ratio (AOR), 0.54–0.65, all *P* < 0.001), after adjustment of the following prognostic factors: fatal comorbidities (McCabe classification), critical illness (Pitt bacteremia score (PBS) ≥4) on arrival at the ED, polymicrobial bacteremia, extended-spectrum beta-lactamase-producer bacteremia, underlying malignancies or liver cirrhosis, and bacteremia caused by pneumonia or urinary tract infections. The adverse impact of TtAa on 28-day mortality was most evident at the cutoff of 48 hours, as the lowest AOR was identified (0.54, *P* < 0.001). In subgroup analyses, the most evident TtAa cutoff (i.e., the lowest AOR) remained at 48 hours in mildly ill (PBS = 0; AOR 0.47; *P* = 0.04) and moderately ill (PBS = 1–3; AOR 0.55; *P* = 0.02) patients, but shifted to 1 hour in critically ill patients (PBS ≥4; AOR 0.56; *P* < 0.001).

**Conclusions:**

The time from triage to administration of appropriate antimicrobials is one of the primary determinants of mortality. The optimum timing of appropriate antimicrobial administration is the first 48 hours after non-critically ill patients arrive at the ED. As bacteremia severity increases, effective antimicrobial therapy should be empirically prescribed within 1 hour after critically ill patients arrive at the ED.

## Background

Despite improvements in hemodynamic support and antibiotic therapy, bacteremia is still associated with high morbidity and mortality, which contribute substantially to healthcare costs [1]. Community-onset bacteremia, with an annual incidence of 0.82% in a population-based study [2], is a common problem for clinicians. In addition to early establishment of vascular access, fluid resuscitation, and removal of infection source, particularly in critically ill patients, antimicrobial therapy has been regarded as one of the mainstays of bacteremia treatment [3]. Although most investigations have concluded that early administration of appropriate antimicrobials could be linked to a favorable prognosis in the individuals with bacteremia [4–7], several studies have not identified the survival benefit of early appropriate antimicrobial therapy [8, 9]. Furthermore, the timing of the first dose of appropriate antibiotics remains a controversial point, and thus appropriate empirical antimicrobial therapy has been defined as the interval between bacteremia onset and antimicrobial administration, ranging widely from <24 hours to <48 hours in previous reports [7, 10, 11]. To our knowledge, the Surviving Sepsis Campaign (SCC) “International guidelines for the management of severe sepsis and septic shock” recommend that an appropriate antimicrobial therapy be administered within the first 1 hour of recognition of severe sepsis or septic shock [3]. However, focusing on patients with bacteremia, the question “what priority is early enough for appropriate antibiotic administration?” has been debated. Therefore, to describe the beneficial effects of appropriate empirical antimicrobial administration on clinical outcome and to further achieve the “optimum timing” of appropriate antimicrobial administration, we compared the outcome impact of different time cutoffs from emergence department (ED) triage to appropriate antimicrobial administration for adults with community-onset bacteremia.

## Methods

### Study design

This retrospective cohort study was conducted from January 2008 to December 2013 at the ED of a medical center in southern Taiwan. The study hospital is a 1200-bed, university-affiliated medical center with an annual ED census of approximate 70,000 patients. The study was reported by the format recommended by the Strengthening the Reporting of Observational Studies in Epidemiology (STROBE) statement; partial clinical information on this study cohort has been published [10–13].

During the study period, the blood cultures sampled at the ED were screened for bacterial growth by a computer database. Clinical information on adults with bacteremia was retrieved from medical charts. In the patients with multiple episodes of bacteremia, only the first episode of each patient was included. Patients were excluded if they had hospital-onset bacteremia, contaminated blood cultures, bacteremia diagnosed prior to visiting the ED, inappropriate dosage or route of antimicrobial administration during all hospitalization, or incomplete clinical information.

### Data collection

A predetermined form was used to collect demographic and clinical characteristics, including age, vital signs on arrival at the ED, comorbidities, type of antimicrobial agents administered and duration of administration, bacteremia source, length of hospital stay, bacteremia severity (Pitt bacteremia score (PBS)) on arrival at the ED, comorbidity severity (McCabe classification), and patient outcome. Of all eligible patients, information was retrieved by retrospective review of medical charts, which was done by two authors, and any discrepancy was resolved by discussion between the authors. The primary endpoint was crude mortality within 28 days after onset of bacteremia (arrival at the ED).

### Definitions

Community-onset bacteremia indicates that the place of onset of the episode of bacteremia is the community, which includes long-term healthcare facility-acquired and community-acquired bacteremia, as previously described [11, 14]. Polymicrobial bacteremia was defined as the isolation of more than one microbial species from a single bacteremia episode. Blood culture samples with potentially contaminating pathogens were considered to be contaminated in accordance with the previous criteria [15].

As previously described [10, 11], the antimicrobial therapy was considered appropriate when all the following criteria were fulfilled: (1) the route and dosage of antimicrobial agents were administered as recommended in the Sanford Guide [16] and (2) the bacteremia pathogens were susceptible in vitro to the administrated antimicrobial agent based on the contemporary breakpoints of the Clinical and Laboratory Standards Institute (CLSI) [17]. The time to appropriate antibiotic (TtAa) measured in hours was defined as the period between the arrival at the ED (i.e., ED triage) and the administration of appropriate antimicrobials [18]. The bacteremia severity was graded according to the PBS using a previously validated scoring system based on vital signs, use of inotropic agents, mental status, receipt of mechanical ventilation, and cardiac arrest [11, 14]. Comorbidities were defined as described previously [19] and malignancies included hematological malignancies and solid tumors. The prognosis of preexisting diseases was assessed by a previous delineated classification system (McCabe classification) [20]. Bacteremia sources were determined clinically on the basis of the previous definitions [11]. Crude mortality was used to define death from all causes.

### Microbiological methods

Blood cultures were incubated in a BACTEC 9240 instrument (Becton Dickinson Diagnostic Systems, Sparks, MD, USA) for 5 days at 35 °C. Bacteremia aerobic isolates were prospectively collected during the study period. The bacterial species was identified by means of the Vitek 2 system (bioMérieux, Durham, NC, USA), and antimicrobial susceptibility was determined by the disk diffusion method, based on the performance standards of CLSI in 2016 [17]. The tested drugs included ampicillin/sulbactam, piperacillin/tazobactam, cefazolin, cefuroxime, cefotaxime, ceftazidime, cefepime, ertapenem, imipenem, and levofloxacin. If the patient was empirically treated by other agents, the susceptibility of the indicated agent was measured. For *Escherichia coli, Klebsiella* species, and *Proteus mirabilis* (EKP)*,* extended-spectrum beta-lactamase (ESBL) production was detected by the phenotypic confirmatory test with the cephalosporin-clavulanate combination disks recommended by the previous CLSI guidelines in 2009 [21].

### Statistical analyses

Statistical analyses were performed using the Statistical Package for the Social Sciences for Windows (Version 20.0; Chicago, IL, USA). Clinical data, demographic data, severity, and patient outcomes were compared using the Fisher exact test or the Pearson chi-square test for categorical variables and the independent samples *t* test or the Mann–Whitney *U* test for continuous variables. To assess the independent predictors with adjusted odds ratios (AORs), the variables in the univariate analysis with a *P* value <0.05 were included in a stepwise and backward multivariable logistic regression model. Several TtAa cutoffs were selected: 1 hour*,* 6 hours, 12 hours, 24 hours, 48 hours, 72 hours, and 96 hours. Cox regression was used to study the impact of varied TtAa cutoffs on clinical outcomes after adjustment for independent risk factors for 28-day mortality.

As all available data were used to maximize the power, no sample size calculation was performed. As suggested, at least 8–10 events per variable are required for reliable multiple logistic regression analysis [22]. For missing data, a complete case analysis was conducted if the percentage of missing values was <5%. If the percentage of missing values was ≥5%, appropriate imputation was performed [23]. A two-tailed *P* value <0.05 was considered significant.

## Results

### Demographics and clinical characteristics of the patient cohort

Of 3934 patients with bacterial growth in blood cultures, 2349 adults with community-onset bacteremia were analyzed, after exclusion of 1503 adults with contaminated blood cultures, 66 with hospital-onset bacteremia or with bacteremia prior to arrival at the ED, 13 with an uncertain outcome within 28 days of onset of bacteremia, and 3 with inappropriate dosage or route of antimicrobial administration during all hospitalization. Among the 2349 eligible patients the mean age was 67.9 years and 1029 patients (51.5%) were male. The ten most common sources of bacteremia were urinary tract infections (748 patients, 31.1%), pneumonia (325 patients, 13.5%), intra-abdominal infections (312 patients, 13.0%), skin and soft-tissue infections (244 patients, 10.1%), biliary tract infections (220 patients, 9.2%), primary bacteremia (191 patients, 7.9%), bone and joint infections (92 patients, 3.8%), vascular-line infections (90 patients, 3.7%), liver abscess (79 patients, 3.3%), and infective endocarditis (71 patients, 3.0%).

The mean (interquartile range) length of ED stay was 19.6 (5.3–25.6) hours. Among the patients, 1829 (77.9%) were admitted to general wards and 324 (13.8%) to intensive care units (ICUs). The mean (interquartile range) length of hospital stay was 14.9 (6.1–17.0) days. There were 77 patients (3.3%) who died during the ED stay. There were 119 (5.1%) patients discharged from the ED and followed up in the outpatient clinics. The proportion of critically ill (PBS ≥4) and mildly ill (PBS = 0) patients at ED arrival was 20.3% (476 patients) and 26.9% (633), respectively. The overall 28-day crude mortality rate was 14.5% (340 patients).

### Baseline characteristics of fatal and surviving patients

Univariate analyses were used to compare clinical variables among adults with fatal and surviving outcome (Table 1). Nursing-home residents, male gender, critical illness (PBS ≥4) on arrival at the ED, initial syndromes of severe sepsis or septic shock on arrival at the ED, fatal comorbidities (McCabe classification), polymicrobial bacteremia, *K. pneumoniae*, *Pseudomonas* species, or ESBL-producing EKP bacteremia, bacteremia pneumonia, underlying neurological diseases or liver cirrhosis, thrombocytopenia, or elevated serum levels of C-reactive protein were more frequently noted in patients who died. In contrast, *E. coli* bacteremia, bacteremia due to urinary tract infections, biliary tract infections, or liver abscess, or underlying hypertension, were more often present in survivors at 28 days after bacteremia onset.

**Table 1:** Patient demography, bacteremia severity, comorbidity severity, causative microorganisms, and laboratory data for adults with community-onset bacteremia, categorized by 28-day crude mortality

Variable	28-day Mortality, number of cases (%)		*P* value
	Yes (*n* = 340)	No (*n* = 2009)	
Age ≥65 years	223 (65.6)	1,227 (61.1)	0.11
Gender, male	205 (60.3)	1,004 (50.0)	<0.001
Nursing-home residents	42 (12.4)	91 (4.5)	<0.001
Severity-of-illness markers at arrival in the ED			
Pitt bacteremia score ≥4	217 (63.8)	259 (12.9)	<0.001
Initial sepsis-related syndrome			
Severe sepsis	289 (85.0)	734 (36.5)	<0.001
Septic shock	225 (66.2)	254 (12.6)	<0.001
Ultimately and rapidly fatal comorbidity (McCabe classification)	159 (46.8)	405 (20.2)	<0.001
Polymicrobial bacteremia	62 (18.2)	178 (8.9)	<0.001
Major causative microorganisms			
*Escherichia coli*	88 (25.9)	880 (43.8)	<0.001
*Klebsiella pneumoniae*	77 (22.6)	277 (13.8)	<0.001
*Staphylococcus aureus*	53 (15.6)	259 (12.9)	0.18
*Streptococcus* species	45 (13.2)	237 (11.8)	0.45
*Pseudomonas* species	28 (8.2)	61 (3.0)	<0.001
ESBL-producing EKP	27 (7.9)	52 (2.6)	<0.001
Major bacteremia sources			
Pneumonia	120 (35.3)	205 (10.2)	<0.001
Urinary tract infection	39 (11.5)	709 (35.3)	<0.001
Biliary tract infection	20 (5.9)	200 (10.0)	0.02
Intra-abdominal infection	47 (13.8)	265 (13.2)	0.75
Liver abscess	4 (1.2)	75 (3.7)	0.02
Skin and soft-tissue infection	36 (10.6)	208 (10.4)	0.90
Primary bacteremia	31 (9.1)	150 (7.5)	0.29
Major comorbidities			
Malignancy	159 (46.8)	500 (24.9)	<0.001
Hypertension	140 (41.2)	978 (48.7)	0.01
Diabetes mellitus	117 (34.4)	758 (37.7)	0.24
Chronic kidney disease	58 (17.1)	361 (18.0)	0.69
Neurological disease	95 (27.9)	427 (21.3)	0.006
Liver cirrhosis	70 (20.6)	231 (11.5)	<0.001
Laboratory data on arrival at the ED			
Leukocytes (10^3^/mm^3^)	14.8 ± 25.7	12.5 ± 7.2	0.11
Platelet (10^3^/mm^3^)	162.4 ± 123.1	199.4 ± 114.4	<0.001
Serum creatinine (mg/dL)	2.4 ± 3.0	2.2 ± 15.3	0.76
C-reactive protein (mg/L)	162.3 ± 147.5	113.8 ± 118.9	<0.001

*ED* emergency department, *ESBL* extended-spectrum beta-lactamase, *EKP Escherichia coli, Klebsiella* species*,* and *Proteus mirabilis*

All variables are expressed as number of cases (%), but laboratory data as mean ± standard deviation

### Bacteremia isolates and susceptibility

Because of 240 (10.2%) events of polymicrobial bacteremia and 2109 monomicrobial bacteremia, a total of 2655 causative microorganisms from 2349 patients were collected. The ten leading microorganisms included *E. coli* (968, 36.5%), *Klebsiella* species (371, 14.0%), *Staphylococcus aureus* (312, 11.8%), *Streptococcus* species (289, 10.9%), *Pseudomonas* species (89, 3.4%), *Enterococcus* species (88, 3.3%), *Proteus* species (68, 2.6%), *Salmonella* species (59, 2.2%), *Aeromonas* species (45, 1.7%), and *Enterobacter* species (39, 1.5%). Overall, cefazolin or cefuroxime was active against 7.6–78.0% or 15.4–89.3% of varied Gram-negative aerobes, respectively. Levofloxacin, cefotaxime, ceftazidime, cefepime, or ertapenem was active against 84.3–100%, 86.1–96.6%, 86.1–96.6%, 94.4–100%, or 96.8–100%, respectively, of Gram-negative species, and imipenem was active against all Gram-negative aerobes. ESBL production was detected in 5.6% (79) of 1401 EKP isolates. Of 312 *S. aureus* isolates, 37.2% (116 isolates) were resistant to methicillin.

### Predictors of 28-day mortality

The association between 28-day crude mortality and clinical variables, including age, sex, bacteremia severity, bacteremia source, comorbidity severity, causative microorganisms, and major comorbidities was examined by univariate analysis of 2349 adults. The following variables were positively associated with 28-day mortality: having male gender (odds ratio (OR) 1.52; *P* < 0.001), being resident in a nursing home (OR 2.97; *P* < 0.001), having ultimately or rapidly fatal comorbidities (McCabe classification; OR 3.48; *P* < 0.001), being critically ill (PBS ≥4; OR 11.92; *P* < 0.001) on arrival at the ED, having polymicrobial bacteremia (OR 2.29; *P* < 0.001), *K. pneumoniae* (OR 1.83; *P* < 0.001), *Pseudomonas* species (OR 2.87; *P* < 0.001), or ESBL-producing EKP (OR 3.25; *P* < 0.001) bacteremia, having bacteremia pneumonia (OR 4.80; *P* < 0.001), and having comorbidities with malignancies (OR 2.65; *P* < 0.001), neurological diseases (OR 2.00; *P* = 0.006), or liver cirrhosis (OR 1.44; *P* < 0.001). Furthermore, the following variables were negatively associated with 28-day mortality: being mildly ill (PBS = 0; OR 0.23; *P* < 0.001), having *E. coli* bacteremia (OR 0.45; *P* < 0.001), having bacteremia due to urinary tract infections (OR 0.24; *P* < 0.001), having biliary tract infection (OR, 0.57; *P* = 0.02) or liver abscess (OR 0.31; *P* = 0.02), and having comorbidities with hypertension (OR 0.74; *P* = 0.01). However, eight independent predictors were identified in the multivariate regression analysis (Table 2): being critically ill (PBS ≥4) on arrival at the ED, having ultimately or rapidly fatal comorbidities (McCabe classification), polymicrobial bacteremia, ESBL-producing EKP bacteremia, or bacteremia pneumonia, and preexisting malignancy or liver cirrhosis. One negative predictor was the presence of urinary tract infection as the source of bacteremia.

**Table 2:** Independent predictors of 28-day mortality in 2349 adults with community-onset bacteremia

Variables	AOR (95% CI)	*P* value
Critical illness (Pitt bacteremia score ≥4) on arrival at the ED	8.77 (6.43–11.97)	<0.001
Ultimately and rapidly fatal comorbidity (McCabe classification)	2.21 (1.48–2.92)	<0.001
Polymicrobial bacteremia	2.00 (1.35–2.97)	0.001
ESBL-producing EKP bacteremia	6.00 (3.11–11.55)	<0.001
Bacteremia source		
Pneumonia	2.08 (1.48–2.92)	<0.001
Urinary tract infection	0.46 (0.25–0.84)	0.01
Comorbidity		
Malignancy	1.81 (1.30–2.50)	<0.001
Liver cirrhosis	3.04 (1.19–3.97)	<0.001

*AOR* adjusted odds ratio, *CI* confidence interval, *ED* emergency department, *ESBL* extended-spectrum beta-lactamase, *EKP Escherichia coli, Klebsiella* species, and *Proteus mirabilis*

### Impact of different TtAa cutoffs

Of 2349 patients with community-onset bacteremia, the mean (interquartile range) TtAa was 2.0 (<1 to 12) hours. The majority (1663 patients, 70.8%) were treated by appropriate antimicrobials within 1 hour of arrival at the ED (Table 3). All selected TtAa cutoffs, ranging from 1 hour to 96 hours, had prognostic significance (*P* < 0.001). In other words*,* those receiving appropriate antibiotics within the cutoff had a lower mortality rate than those treated by appropriate antibiotics after the cutoff time. The associated AORs ranged from 0.54 to 0.65. Of importance, the lowest AOR (0.54) was evidenced in the TaAa cutoff of 48 hours.

**Table 3:** Critical illness, fatal comorbidities, and 28-day mortality rates among adults with community-onset bacteremia

TtAa cutoffs (number of cases)	Percentage (number of cases)		28-day Mortality rate (number of cases)	Univariate analysis		Cox regression	
	Critical illness^a^	Fatal comorbidity		OR (95% CI)	*P* value	AOR (95% CI)^b^	*P* value
1 hour							
≤1 (*n* = 1663)	16.5 (275)	22.4 (373)	10.3 (172)	0.36 (0.28–0.45)	<0.001	0.57 (0.46–0.71)	<0.001
>1 (*n* = 686)	29.3 (201)	27.8 (191)	24.5 (168)				
6 hours							
≤6 (*n* = 1736)	19.0 (329)	23.1 (401)	12.0 (208)	0.50 (0.39–0.63)	<0.001	0.65 (0.51–0.82)	<0.001
>6 (*n* = 613)	24.0 (147)	26.6 (163)	21.5 (132)				
12 hours							
≤12 (*n* = 1760)	19.4 (341)	23.4 (412)	12.3 (216)	0.53 (0.41–0.67)	<0.001	0.65 (0.52–0.83)	<0.001
>12 (*n* = 589)	22.9 (135)	25.8 (152)	21.1 (124)				
24 hours							
≤24 (*n* = 1,853)	19.8 (367)	23.5 (436)	12.6 (234)	0.53 (0.41–0.69)	<0.001	0.62 (0.48–0.79)	<0.001
>24 (*n* = 496)	22.0 (109)	25.8 (128)	21.4 (106)				
48 hours							
≤48 (*n* = 1,917)	19.8 (380)	23.5 (450)	12.5 (240)	0.48 (0.37–0.62)	<0.001	0.54 (0.43–0.71)	<0.001
>48 (*n* = 432)	22.2 (96)	26.4 (114)	23.1 (100)				
72 hours							
≤72 (*n* = 1,981)	19.5 (387)	23.4 (463)	12.6 (249)	0.44 (0.33–0.57)	<0.001	0.55 (0.43–0.71)	<0.001
>72 (*n* = 368)	24.2 (89)	27.4 (101)	24.7 (91)				
96 hours							
≤96 (*n* = 2,061)	19.4 (400)	23.5 (484)	12.8 (264)	0.41 (0.31–0.55)	<0.001	0.56 (0.44–0.72)	<0.001
>96 (*n* = 288)	26.4 (76)	27.8 (80)	26.4 (76)				

The significance of the effects of different cutoffs of the time to appropriate antibiotic (TtAa) on 28-day mortality were examined by univariate and Cox regression analyses. *OR* odds ratio, *CI* confidence interval, *AOR* adjusted odds ratio. ^a^Pitt bacteremia score ≥4 on arrival at the emergency department. ^b^Adjusted for independent predictors of 28-day mortality recognized in the multivariate regression: critical illness; a fatal comorbidity (McCabe classification); polymicrobial bacteremia; Extended-spectrum beta-lactamase-producing bacteremia; bacteremia because of pneumonia or urinary tract infections; and underlying malignancy or liver cirrhosis

### Impact of different TtAa cutoffs on patients with different severity of bacteremia

**Fig. 1:**
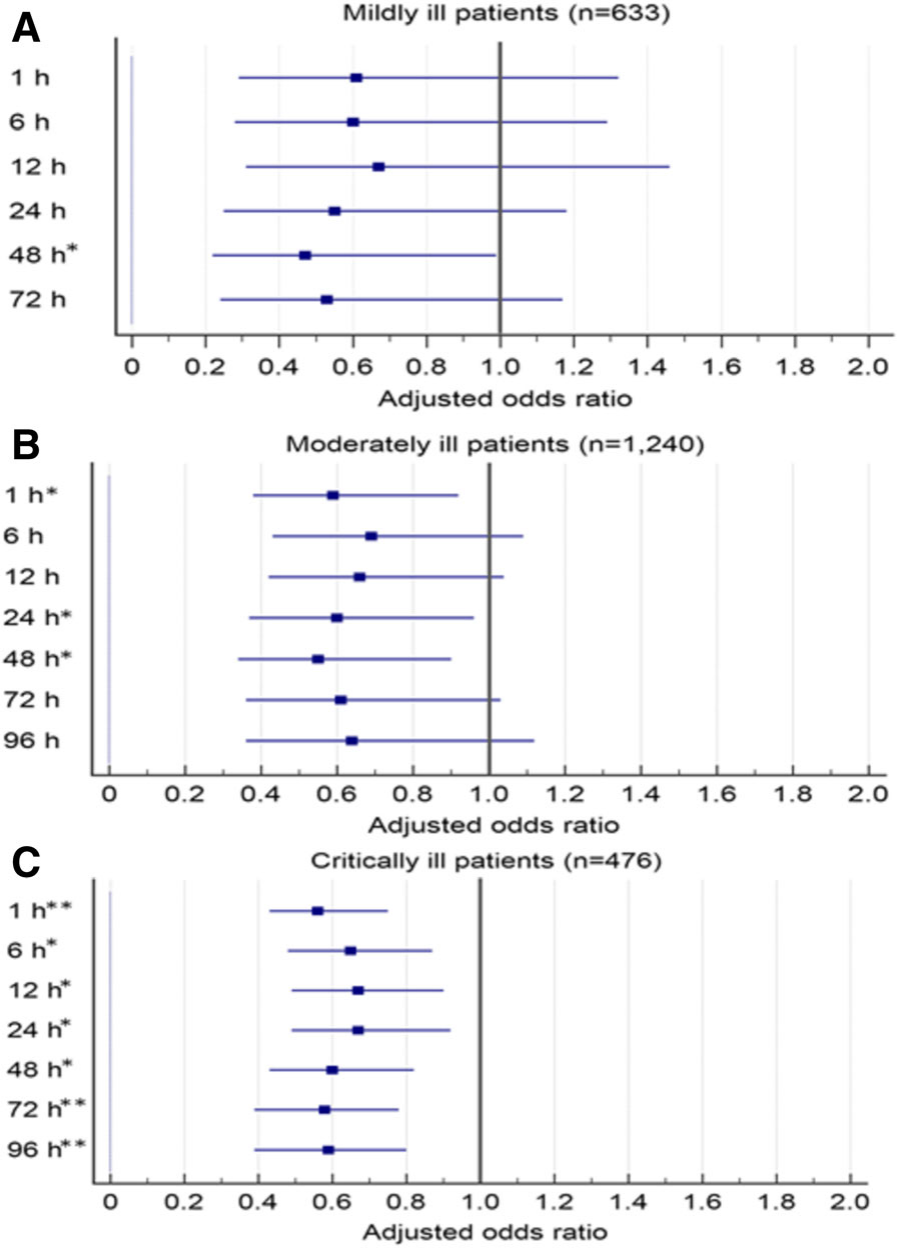
Adjusted odds ratios of different cutoffs of the time-to-appropriate antibiotic for 28-day crude mortality in adults, categorized by the Pitt bacteremia score on arrival at the emergency department in mildly ill (**a**), moderately ill (**b**), and critically ill (**c**) patients, using Cox regression after adjustment for the independent predictors of 28-day mortality (including a fatal comorbidity (McCabe classification), polymicrobial bacteremia, extended-spectrum beta-lactamase-producing bacteremia, bacteremia because of pneumonia or urinary tract infections, and underlying malignancies or liver cirrhosis). *P value <0.05; **P value <0.001

According to the severity of bacteremia on arrival at the ED, all patients were categorized into three groups: mildly ill (PBS = 0; 633 patients, 26.9%), moderately ill (PBS = 1–3; 1240 patients, 52.8%), and critically ill (PBS ≥4; 476 patients, 20.2%), as shown in Table 4. In mildly ill patients, after adjustment of independent predictors of 28-day mortality, only the TtAa cutoff of 48 hours (AOR = 0.47; *P* = 0.04) was significantly associated with 28-day mortality (Fig. 1a). Similarly, in moderately ill patients, three TtAa cutoffs (1 hour, 24 hours, and 48 hours) were significantly related to 28-day mortality, and the lowest AOR (0.55) was noted for the TtAa cutoff of 48 hours (Fig. 1b). Focusing on critically ill patients, all TtAa cutoffs were linked to 28-day mortality and the lowest AOR (0.56) was identified for the TtAa cutoff of 1 hour (Fig. 1c).

**Table 4:** Impact of different cutoffs for the time-to-appropriate antibiotic (TtAa) on 28-day crude mortality in adults categorized by Pitt bacteremia score (PBS) on arrival at the emergency department

TtAa (hours)	Mortality rate (%)		Univariate analysis	
	≤ TtAa	> TtAa	OR (95% CI)	*P* value
Mildly ill (PBS = 0, *n* = 633)				
1	3.6	8.4	0.41 (0.20–0.85)	0.01
6	3.6	8.8	0.39 (0.19–0.80)	0.008
12	3.8	8.3	0.43 (0.21–0.90)	0.02
24	3.7	9.1	0.38 (0.18–0.80)	0.008
48	3.6	10.2	0.33 (0.16–0.69)	0.002
72	3.8	10.5	0.34 (0.16–0.73)	0.004
96	4.4	8.5	0.48 (0.20–1.17)	0.10
Moderately ill (PSB = 1–3, *n* = 1240)				
1	6.0	11.6	0.48 (0.31–0.75)	0.001
6	6.3	10.7	0.56 (0.36–0.88)	0.01
12	6.3	11.1	0.54 (0.34–0.84)	0.006
24	6.3	11.9	0.50 (0.32–0.80)	0.003
48	6.3	13.0	0.45 (0.28–0.73)	0.001
72	6.6	12.6	0.49 (0.29–0.81)	0.005
96	6.8	13.1	0.48 (0.28–0.84)	0.009
Critical ill (PBS ≥4, *n* = 476)				
1	36.4	58.2	0.41 (0.28–0.60)	<0.001
6	40.1	57.8	0.49 (0.33–0.73)	<0.001
12	40.8	57.8	0.50 (0.34–0.75)	0.001
24	41.7	58.7	0.50 (0.33–0.78)	0.002
48	41.3	62.5	0.42 (0.27–0.67)	<0.001
72	41.1	62.4	0.43 (0.23–0.60)	<0.001
96	41.2	62.5	0.43 (0.24–0.62)	<0.001

*OR* odds ratio, *CI* confidence interval

## Discussion

Bacteremia is associated with substantial morbidity and mortality and early interventions are often emphasized [1, 14]. However, the estimates of potential benefits of appropriate empirical antibiotic treatment vary widely in the literature, from no effect [8, 9] to significant reduction in fatality with an odds ratio of up to 6 [4–7]. Such a controversy may be related to variations in sepsis severity, comorbidities and the immune status of study cohorts, and the distribution of causative microorganisms [24]. Similar to previous reports dealing with the crucial relationship between patient outcomes and the appropriateness of empirical antimicrobial therapy for specific pathogens, such as Gram-negative bacilli [25], *E. coli* [26, 27], *K. pneumoniae* [27], *S. aureus* [28], or bloodstream infections [5, 6], our results support that appropriate empirical antimicrobial therapy reduces short-term mortality in patients with community-onset bacteremia. Of importance, in accordance with increasing bacteremia severity, appropriate antimicrobials should be given as soon as possible.

Several investigations have attempted to answer the question “How early is enough to administer appropriate antimicrobials in patients with severe infection?” However, different TtAa cutoffs were determined in heterogeneous patient populations reported in the literature. First, for patients with severe *Legionella pneumophila* pneumonia*,* delays >8 hours between appropriate antimicrobial administration and ICU admission resulted in increased mortality [29]. Second, in another study of patients with cancer and septic shock, a delay of >2 hours from ICU admission was associated with a poor prognosis [30]. Finally, in a study of ED patients with severe sepsis and septic shock, a TtAa <1 hour led to a better short-term survival rate [18].

To our knowledge, the present study was the first one focusing on community-onset bacteremia to prioritize antimicrobial therapy in the initial therapeutic strategy. Similar to a previous report of hospital-based bloodstream infection [7], the “optimum timing” of appropriate antimicrobial administration was determined as the first 48 hours after arrival at the ED. Furthermore, consistent with the SSC recommendations [3], this time should be shortened to 1 hour in critically ill patients. However, the administration of in vitro effective antimicrobials to treat bloodstream infections should be as early as possible to achieve optimal treatment of bacterial infections. Any delay in the administration of appropriate drugs should be avoided, if there is a suspicion of severe sepsis or septic shock.

This study has several limitations. First, antimicrobial therapy is one of the cornerstones of therapeutic strategies for patients with bloodstream infection. Nevertheless, for critically ill patients with the complications of severe sepsis and septic shock, other elements of early goal-directed therapy, such as central venous pressure, mean arterial pressure, and central venous oxygen saturation, were not evaluated as covariates in this study. Second, in the assessment of the primary endpoint and analysis of the impact of antimicrobial therapy, we excluded patients with incomplete clinical information and those receiving inappropriate antibiotic dosage or route of administration. As a result, only 16 patients were excluded and thus the selection bias was expected to be minimal. Third, as previous investigations dealing with the effects of the appropriateness of empirical antimicrobial administration on patient outcome [4–9], no detailed information was available in our study on early resuscitation. However, the majority (1663, 70.8%) of the 2349 patients with community-onset bacteremia received the first dose of appropriate antimicrobials within one hour of arrival at the ED, and the study population were believed to be managed according to the SSC recommendations. Thus, the clinical outcomes of the patients receiving appropriate antimicrobial therapy at different time points after arrival at the ED could be compared with each other. Fourth, as noted before [4–9], no analyses of source control were performed in the present study. However, taking the cases of urosepsis as the example, in those with or without interventions for source control, such as percutaneous nephrolithotomy or nephrectomy, the 28-day crude mortality rate was not different (4/48, 8.3% vs. 35/700, 5.0%; *P* = 0.31), indicative of standard quality of clinical care in the study hospital. Finally, as this study was conducted at one hospital, the critical TtAa cutoffs we identified may not be generalizable to other communities with variable infection sources or severity of community-onset bacteremia. However, our findings highlight that more severely ill patients with bacteremia require earlier administration of appropriate antimicrobials to achieve a favorable outcome.

## Conclusions

Our results demonstrate that the elapsed time from arrival at the ED to the administration of appropriate antimicrobials is a crucial determinant of short-term outcomes in patients with community-onset bacteremia. The optimum timing of appropriate antimicrobial administration in mildly ill patients was the first 48 hours after arrival at the ED. With increasing severity of bacteremia, this time should be limited to the first 1 hour in critically ill patients. Therefore, to achieve a favorable outcome in critically ill patients, epidemiological surveillance, rapid pathogen identification, or the incorporation of broad-spectrum antimicrobials as empirical therapy into an antibiotic stewardship program should be considered.

## Abbreviations

AOR: Adjusted odds ratio
CI: Confidence interval
CLSI: Clinical and Laboratory Standards Institute
ED: Emergency department
EKP: *Escherichia coli, Klebsiella* species*,* and *Proteus mirabilis*
ESBL: Extended-spectrum beta-lactamase
ICUs: Intensive care units
OR: Odds ratio
PBS: Pitt bacteremia score
SSC: Surviving Sepsis Campaign
TtAa: Time to appropriate antibiotic
